# An Acute Large Pericardial Effusion in a Scleroderma Renal Crisis

**DOI:** 10.7759/cureus.41897

**Published:** 2023-07-14

**Authors:** Muhammad Ghallab, Hazem Abosheaishaa, Muhammad Haseeb ul Rasool, Nicole C Noff, Daniel Miller, Mahmoud Alashry, Asma Hosna, Giovina Collura

**Affiliations:** 1 Internal Medicine, Icahn School of Medicine at Mount Sinai, New York City (NYC) Health and Hospitals, New York, USA; 2 Internal Medicine/Gastroenterology, Cairo University, Cairo, EGY; 3 Cardiology, Icahn School of Medicine at Mount Sinai, New York City (NYC) Health and Hospitals, New York, USA

**Keywords:** cardiology, sclerodermal renal crisis, impending cardiac tamponade, pericardial effusion, scleroderma

## Abstract

This case report describes a patient with systemic sclerosis (SSc) who presented with a large pericardial effusion and a scleroderma renal crisis (SRC). The patient's clinical presentation, diagnostic workup, and treatment plans are reviewed. The coexistence of these complications presents a challenging clinical scenario requiring an interdisciplinary approach. The management of pericardial effusion in SSc and SRC is discussed, emphasizing the need for early detection and prompt treatment. Further research is needed to better understand and manage these complex complications in SSc patients.

## Introduction

Scleroderma renal crisis (SRC) is a severe complication of systemic sclerosis (SSc) characterized by malignant hypertension and rapidly progressive renal failure. SRC is linked to high morbidity and mortality; therefore, early detection and prompt treatment are essential for the best possible patient outcomes [1]. SSc can also impact the cardiovascular system, several other organ systems, and the kidneys. Pericardial involvement is not uncommon and usually presents with pericardial inflammation. Pericardial effusion is an uncommon presentation and might occur secondary to pericardial inflammation or poor pericardial fluid absorption. A significant pericardial effusion may end up in cardiac tamponade, a potentially fatal condition that needs prompt drainage [2]. It presents a difficult clinical challenge to treat the renal and cardiac manifestations of SSc when an SRC and a significant pericardial effusion coexist.

This case report aims to describe an SSc patient who presented with a significant pericardial effusion and an SRC. We will review this patient's clinical presentation, diagnostic workup, and treatment plans. In addition, we will review the most recent research on managing pericardial effusion in SSc and SRC, emphasizing the significance of an interdisciplinary approach in managing such complex cases. Further research and comprehension are required because few studies specifically address this particular set of complications in SSc patients.

## Case presentation

**Figure 1:**
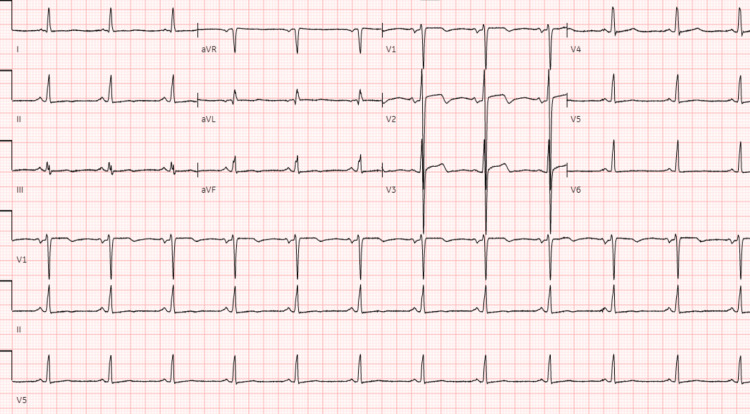
Twelve leads EKG showing biphasic T waves in V2 and V3, along with non-specific ST-T wave changes in inferior and lateral leads

A 70-year-old female with a past medical history of diabetes, hypertension, hyperlipidemia, gastroesophageal reflux disease, SSc, history of provoked deep vein thrombosis with pulmonary embolism requiring placement of inferior vena cava filter presented to the emergency department with altered mental status, chest pain, and right arm weakness for three days. She was diagnosed with SSc seven years prior and started on methotrexate. Later, the patient stopped taking methotrexate on her own, complicating the disease course with the development of esophageal dysmotility and skin thickening. Patients' home medications include atorvastatin, carvedilol, promethazine, and sucralfate. At the time of presentation, the patient's blood pressure was 239/159 mmHg, heart rate was 88 per minute, and respiratory rate was 26 breaths per minute. On the physical examination, the patient was non-cooperative and confused. The cardiovascular and pulmonary examinations were unremarkable. Distal pulses were equal and comparable bilaterally. CT head and CT angiography of the head and neck were performed, which were suggestive of atrophy and sequela of prior small vessel ischemia, with no acute intracranial abnormality. EKG revealed biphasic T waves in V2 and V3, along with non-specific ST-T wave changes in the inferior and lateral leads (Figure 1). Troponin-T was elevated on admission, and the repeat increased even more, but by the third blood draw, it had decreased slightly. The troponin was likely elevated due to the hypertensive emergency.

The cardiology service was consulted and they recommended starting labetalol and doing serial EKGs for possible signs of ischemia. The neurology service was also consulted and recommended to control the blood pressure. Despite receiving oral and parenteral labetalol for blood pressure control, there was a minimal change in blood pressure. The patient was transferred to the ICU for intravenous nicardipine infusion, which resulted in blood pressure control. Due to concern for SRC, thrombotic microangiopathy, and microangiopathic hemolytic anemia, a peripheral smear, G6PD levels, and ADAMTS13 were requested. Peripheral smear revealed a moderate percentage of schistocytes. Low-density lipoprotein (LDL) was elevated (Table 1). Reticulocyte count was within normal limits (Table 1). Haptoglobin was low (Table 1), which is consistent with the diagnosis of intravascular hemolysis. ADAMTS13 level was normal. Her renal functions started to decline rapidly, with creatinine rising from baseline and nearly doubling (Table 1). Due to deteriorating renal function, the patient was started on an angiotensin-converting enzyme inhibitor (ACE-I) Infusion.

**Table 1:** Lab results LDL: low-density lipoprotein

Lab test	Result at presentation	Result at first follow-up labs	Result at second follow-up labs	Reference range
Troponin-T	0.349 ng/ml	0.404 ng/ml	0.377 ng/ml	<0.010 ng/ml
LDL	495 U/L	-	-	135-214 U/L
Reticulocytes	0.0589x10(6)/mcL	-	-	0.0221-0.0963 x10(6)/mcL
Haptoglobin	26 mg/dL	-	-	34-200 mg/dL
Creatinine	3.0 mg/dL	4.7 mg/dL	3.3 mg/dL	0.50-0.90 mg/dL

**Figure 2:**
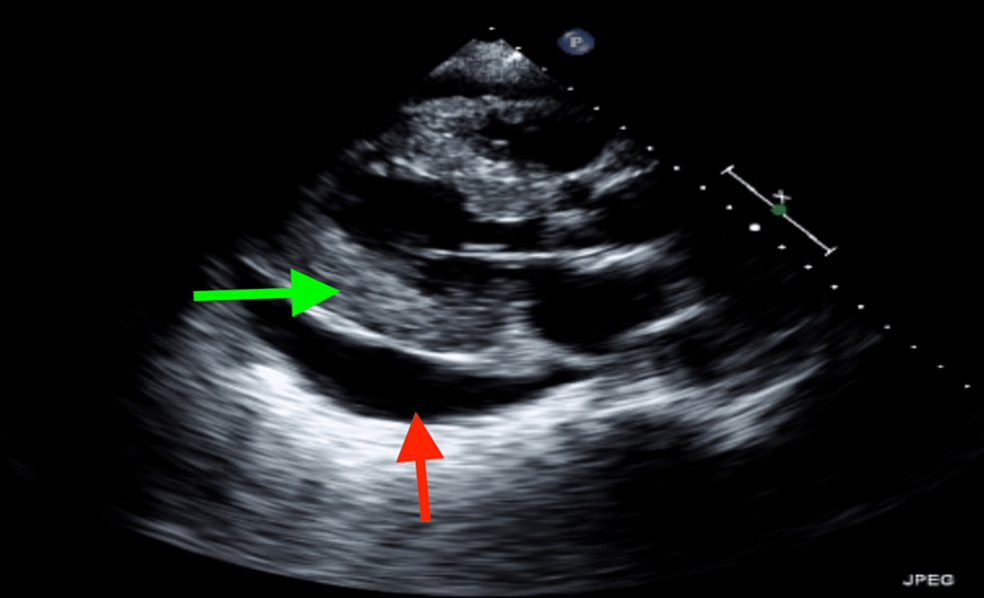
Transthoracic echocardiogram, parasternal long axis view, at the time of admission demonstrating large pericardial effusion at the LV posterior wall and RV free wall and LV hypertrophy The red arrow points to the pericardial effusion. The green arrow points to the left hypertrophied ventricle.

**Figure 3:**
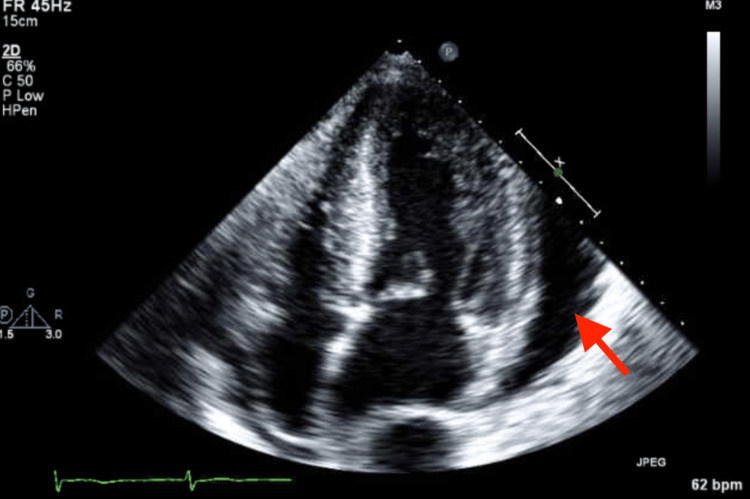
Transthoracic echocardiogram, apical four chambers view, at the time of admission demonstrating large pericardial effusion at the LV lateral wall and RV free wall and increased LV wall thickness The red arrow points to pericardial effusion.

Echocardiogram was performed for suspected acute non-ST segment elevation myocardial infarction, which revealed an increased left ventricle (LV) wall thickness with prominent hypertrophy of the mid-LV segments suggestive of hypertrophic cardiomyopathy or infiltrative cardiomyopathy, hyperdynamic LV contractility, LV ejection fraction of 60%, and large pericardial effusion which was circumferential and measures up to 2.4 cm adjacent to the inferolateral wall of the LV, and the features were not consistent with tamponade physiology, as there was no systolic or diastolic collapse of the atriums (Figures 2, 3, Video 1).

**Video 1:** Transthoracic echocardiogram, apical four chambers view, at the time of admission demonstrating large pericardial effusion at the LV lateral wall and RV free wall with no evidence of tamponade

Evaluation for pulsus paradoxus was positive, raising concern for impending tamponade. A respiratory viral panel was sent due to concern about acute pericardial effusion, but it was negative. An autoimmune workup was within normal apart from an antinuclear antibody titer of 1:2560. Renin activity and aldosterone levels were normal. CT chest and abdomen were unremarkable and had no evidence of malignancy. She was continued on captopril for SRC. The cardiology service evaluated the patient and stated that there were no signs of tamponade and, therefore, no need for pericardiocentesis.

**Figure 4:**
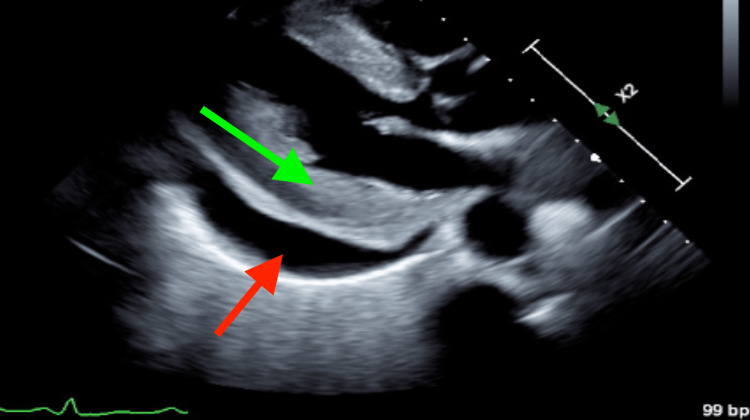
Transthoracic echocardiogram, parasternal long axis view, three weeks after the admission demonstrating stable large pericardial effusion at the LV posterior wall The red arrow points to the pericardial effusion. The green arrow points to the hypertrophied LV.

**Figure 5:**
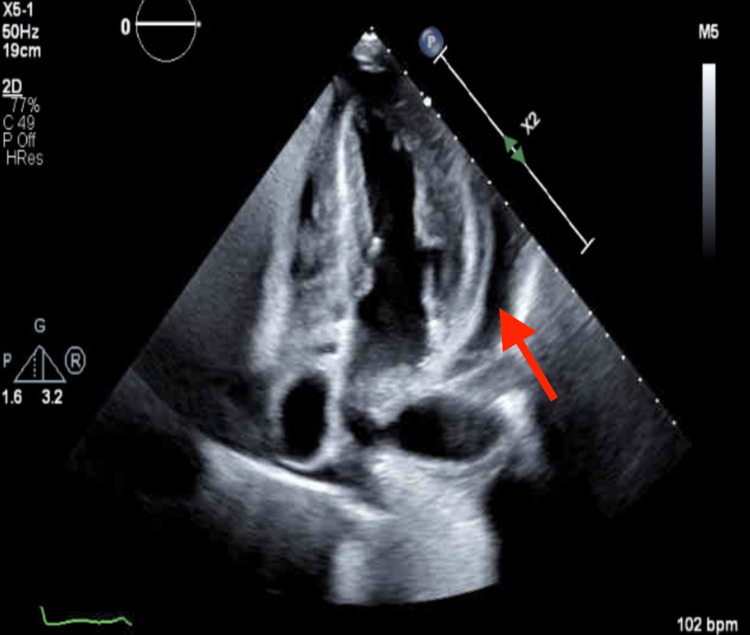
Transthoracic echocardiogram, parasternal long axis view, three weeks after the admission demonstrating stable large pericardial effusion at the LV posterior wall The red arrow points to the pericardial effusion.

The dose of captopril was increased which lead to improved renal function. However, due to poorly controlled blood pressure, she was started on oral amlodipine and nicardipine infusion, which progressively improved blood pressure control. Creatinine peaked and then started to improve (Table 1). Interval echocardiogram revealed a stable large circumferential pericardial effusion with no significant respiratory variations of the transmitral flow and no concerns for physiologic tamponade. There were some organized elements in the effusion near the right ventricular (RV) free wall, RV apex, and LV apex (Figures 4, 5, Video 2).

**Video 2:** Transthoracic echocardiogram, apical four chambers view, three weeks after the admission demonstrating stable large pericardial effusion at the LV lateral wall with right atria free wall buckling but with no evidence of tamponade. There were some organized elements in the effusion near the LV lateral wall

As the posterior effusions are difficult to tap and the malignant workup was negative, the patient was started on a renal-adjusted dose of colchicine and was discharged after improvement of her condition for follow-up in the outpatient clinic and follow-up of the echocardiogram.

## Discussion

SRC occurs in approximately 10% of patients with scleroderma, and progression to end-stage renal disease occurs in 50% of patients [3,4]. Although the crisis is characterized by malignant hypertension and progressive renal failure, 10% of SRC cases present with normotension, termed a normotensive renal crisis. The pathophysiology is thought to be a series of insults to the kidneys yielding endothelial injury, intimal proliferation, and narrowing of renal arterioles resulting in decreased blood flow, hyperplasia of the juxtaglomerular apparatus, hyperreninemia, and accelerated hypertension. Numerous risk factors exist, including rapid skin thickening, specific medications, new-onset microangiopathic hemolytic anemia/thrombocytopenia, cardiac complications, extensive joint contractures, and positive anti-RNA polymerase III antibodies. One of the known risk factors is cardiac complications, including pericardial effusion, congestive heart failure, and arrhythmias [3].

Pericardial involvement in SSc is usually mild and asymptomatic, and symptomatic disease ranges from 7% to 20% of patients with SSc. As the disease progresses, symptoms may begin to develop, such as chest pain or tightness, dyspnea, and fever. Nonetheless, 33% to 72% of autopsy studies in patients with SSc discovered pericardial involvement and 41% in echocardiography. This data signifies the gross underestimation of pericardial disease in SSc. Pericardial involvement consists of pericardial effusion, constrictive pericarditis, and acute pericarditis. Pericardial effusion is the most common, accounting for 15% to 43% of echocardiographic findings and 78% in autopsy studies [5].

Studies now focus on the relationship between renal crisis, pericardial effusion, and pulmonary hypertension [5]. SRC occurs in 4-5% of Japanese patients with SSc and is one of the most severe complications which results in a poor outcome [6]. There are many proposed mechanisms to explain this correlation, including right heart failure diminishing renal cortical perfusion yielding renal ischemia in SSc; however, the actual mechanism is still unknown. Yet, a Japanese study indicated that moderate asymptomatic pericardial effusion may predict early or future renal crises in scleroderma [5]. Factors predicting SRC include diffuse progressive skin thickening, duration of SSc of four years or less, autoantibodies against RNA polymerase III, systemic corticosteroid administration, and recent cardiac events [3].

Regardless of how pericardial effusion and renal crisis interconnect, screening for silent pericardial involvement in SSc is essential due to the poor prognosis if the effusion is present. The primary diagnosis mode should be echocardiography, which will also assess for hemodynamic effects and monitor response to therapy. Although no specific treatment for the pericardial disease in SSc exists, non-steroidal anti-inflammatory therapy with careful monitoring of renal function is encouraged. If cardiac tamponade or pericardial constriction develops, pericardiocentesis or surgical intervention must be considered [5].

With the emergence of angiotensin-converting enzyme inhibitors in the 1970s, mortality associated with SRC decreased from 76% to less than 10%. However, some patients progress to end-stage renal disease and require dialysis and some renal transplantation. While renal transplantation has improved survival, SRC may recur in transplanted kidneys [3].

## Conclusions

SRC and pericardial effusion are serious complications of SSc that require timely diagnosis and intervention. SRC is characterized by rapidly progressive renal failure and malignant hypertension and can lead to significant morbidity and mortality. Pericardial involvement is common in SSc patients, and the relationship between SRC and pericardial effusion is not fully understood. Early detection and regular monitoring of pericardial involvement using echocardiography are crucial for guiding treatment. Managing these complex complications requires an interdisciplinary approach and close collaboration among healthcare specialists. Further research is needed to explore the mechanisms underlying the interplay between pericardial effusion and SRC and to develop targeted treatment strategies.
